# Secondary Cardiac Rhabdomyosarcoma: A Unique Presentation

**DOI:** 10.7759/cureus.104322

**Published:** 2026-02-26

**Authors:** Hibat Allah Kamri, Youssef Daoudi, Oumaima Taoussi, Loubna Hamich, Hajar Rabii, Hicham Benyoussef, Amal El Ouarradi, Said Makani, Nabil Ismaili, Chafik El Kettani, Abdeltif Boulahya, Rachida Habbal, Fatimazahra Merzouk

**Affiliations:** 1 Department of Cardiology, Cheikh Khalifa International University Hospital, Mohammed VI University of Health Sciences (UM6SS), Casablanca, MAR; 2 Department of Oncology, Cheikh Khalifa International University Hospital, Mohammed VI University of Health Sciences (UM6SS), Casablanca, MAR; 3 Department of Cardiovascular Surgery, Cheikh Khalifa International University Hospital, Mohammed VI University of Health Sciences (UM6SS), Casablanca, MAR; 4 Department of Anesthesia and Critical Care, Cheikh Khalifa International University Hospital, Mohammed VI University of Health Sciences (UM6SS), Casablanca, MAR

**Keywords:** cardiac metastasis, cardiac secondary localization, cardiac surgery, cardiac tumor, left atrial mass, metastasis, rhabdomyosarcoma, soft tissue sarcoma

## Abstract

Cardiac tumors are rare and complex, with malignant forms carrying a poor prognosis. These tumors are classified as either primary or secondary, with the latter, including metastases, being more prevalent. Rhabdomyosarcoma, a rare but aggressive soft tissue sarcoma, can occasionally metastasize to the heart. Here, we report the case of a 75-year-old male with metastatic rhabdomyosarcoma originating in the fibular muscles, extending to the left atrium, left ventricle, and bronchi. The patient presented with acute dyspnea, palpitations, and cardiogenic shock. Imaging revealed a large intracardiac mass, and urgent surgical resection was performed, followed by adjuvant chemoradiotherapy. The histopathological examination confirmed the diagnosis of rhabdomyosarcoma. Despite the poor prognosis typically associated with metastatic rhabdomyosarcoma, the patient showed clinical improvement post-surgery and continued treatment. This case highlights the aggressive nature of rhabdomyosarcoma with cardiac involvement and underscores the importance of multimodal management, including surgery and adjuvant therapy, to improve patient outcomes.

## Introduction

Cardiac tumors encompass a heterogeneous and uncommon group of conditions, whose clinical presentation and prognosis vary widely depending on their histological nature and mode of cardiac involvement. They are broadly classified into primary cardiac tumors and secondary cardiac involvement resulting from metastatic disease. While primary cardiac tumors are rare in adults and predominantly benign, with atrial myxoma being the most frequent, secondary cardiac tumors are considerably more common and typically reflect advanced systemic malignancy, conferring a poor prognosis [1,2].

Cardiac metastases most often arise from malignancies such as lung carcinoma, breast cancer, melanoma, and lymphomas, spreading to the heart through hematogenous dissemination, lymphatic extension, or direct invasion. Although frequently underdiagnosed, cardiac involvement may lead to significant clinical consequences, including heart failure, arrhythmias, valvular dysfunction, or systemic embolization, underscoring the importance of timely recognition, particularly in non-cardiology settings [1,3].

Rhabdomyosarcoma is an aggressive soft tissue sarcoma that rarely involves the heart. Among primary malignant cardiac tumors, it represents the second most frequent entity after angiosarcoma; however, secondary cardiac metastases from extracardiac rhabdomyosarcoma remain exceptionally rare. This rarity highlights both the aggressive biological behavior of the tumor and the diagnostic challenges associated with cardiac metastatic disease in this context [2].

We report the case of a 75-year-old male with metastatic rhabdomyosarcoma originating from the fibular musculature, with highly unusual cardiac involvement characterized by left atrial metastasis extending into the left ventricle, as well as bronchial invasion. This case is unique not only because of the exceptional site and pattern of cardiac metastasis but also because it illustrates the complex diagnostic and therapeutic challenges posed by advanced rhabdomyosarcoma with cardiac extension. Through this report, we aim to enhance understanding of the mechanisms, clinical implications, and management considerations of cardiac metastases in rare soft tissue sarcomas [1].

## Case presentation

**Figure 1:**
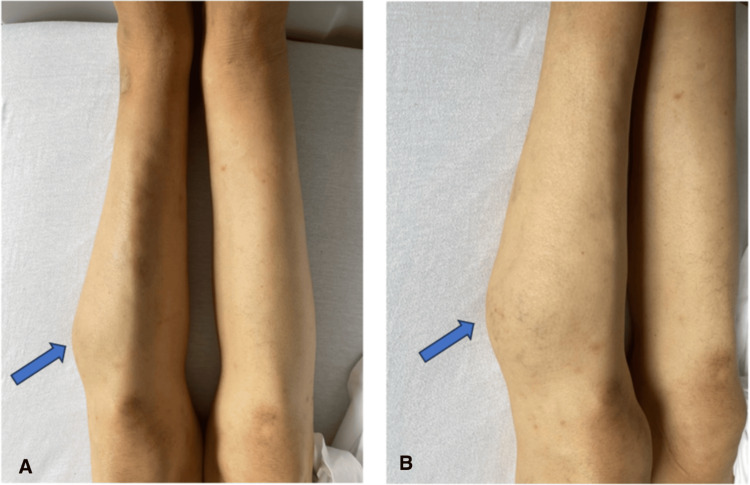
Rounded, firm mass in the upper external third of the left leg (arrow). (A) Frontal view. (B) Lateral view.

A 75-year-old male was admitted to the emergency department with acute-onset exertional and nocturnal dyspnea, classified as New York Heart Association Class III, evolving over the week before admission and associated with nocturnal palpitations. His medical history included atrial fibrillation for eight years, managed with flecainide and rivaroxaban, and hypertension treated with amlodipine. He was also under follow-up for a suspected sarcoma of the left leg, first identified 11 months before admission as a painful, rounded mass in the upper third of the leg (Figure 1), which had progressively increased in size and showed heterogeneous and necrotic components on MRI. Additionally, the patient had no history of toxic exposures and reported neither smoking nor alcohol consumption.

Upon admission to the emergency department, the patient exhibited signs of cardiogenic shock, with hypotension (95/65 mmHg), tachycardia (103 beats/minute), and evidence of peripheral hypoperfusion, including mottling and cold extremities. He also presented with tachypnea at 28 breaths/minute, with an oxygen saturation of 88% on room air, improving to 98% with 5 L/minute of supplemental oxygen. Cardiovascular examination revealed a clearly audible grade 4/6 mitral diastolic rumble with a palpable thrill, and edema of the lower extremities extending to the calves. Osteoarticular assessment identified a well-circumscribed, firm, immobile mass in the upper third of the lateral aspect of the left leg.

Electrocardiography demonstrated atrial fibrillation with a mean ventricular rate of 90 beats/minute and a right bundle branch block. The patient was promptly transferred to the critical care unit for close monitoring and managed with cautious fluid administration, oxygen therapy, and hemodynamic support to stabilize his condition.

**Figure 2:**
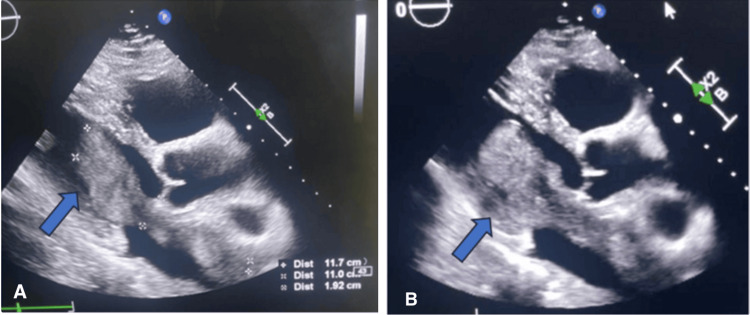
Parasternal long-axis section demonstrating the left atrium mass prolapsing into the left ventricle during diastole measuring 192 × 31 mm (arrow). (A) Parasternal long-axis section demonstrating the dimensions of the left atrial mass prolapsing into the left ventricle. (B) Ventricular implantation of the mass originating from the left atrium.

**Figure 3:**
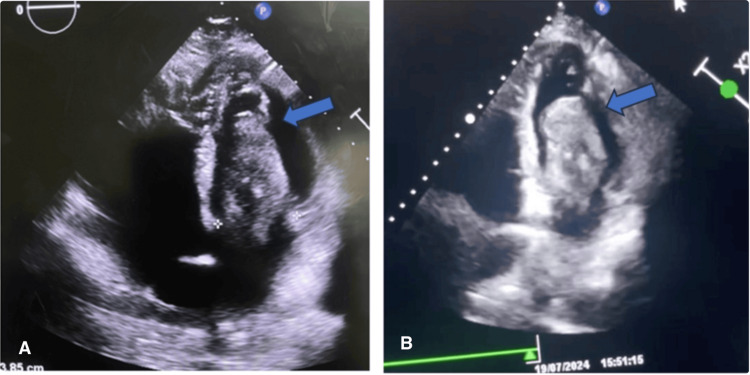
Apical four-chamber view illustrating the extension of the mass into the left ventricle. (A) Apical four-chamber view demonstrating mitral annulus dilation. (B) Apical four-chamber view depicting the appearance of the left ventricular mass.

**Figure 4:**
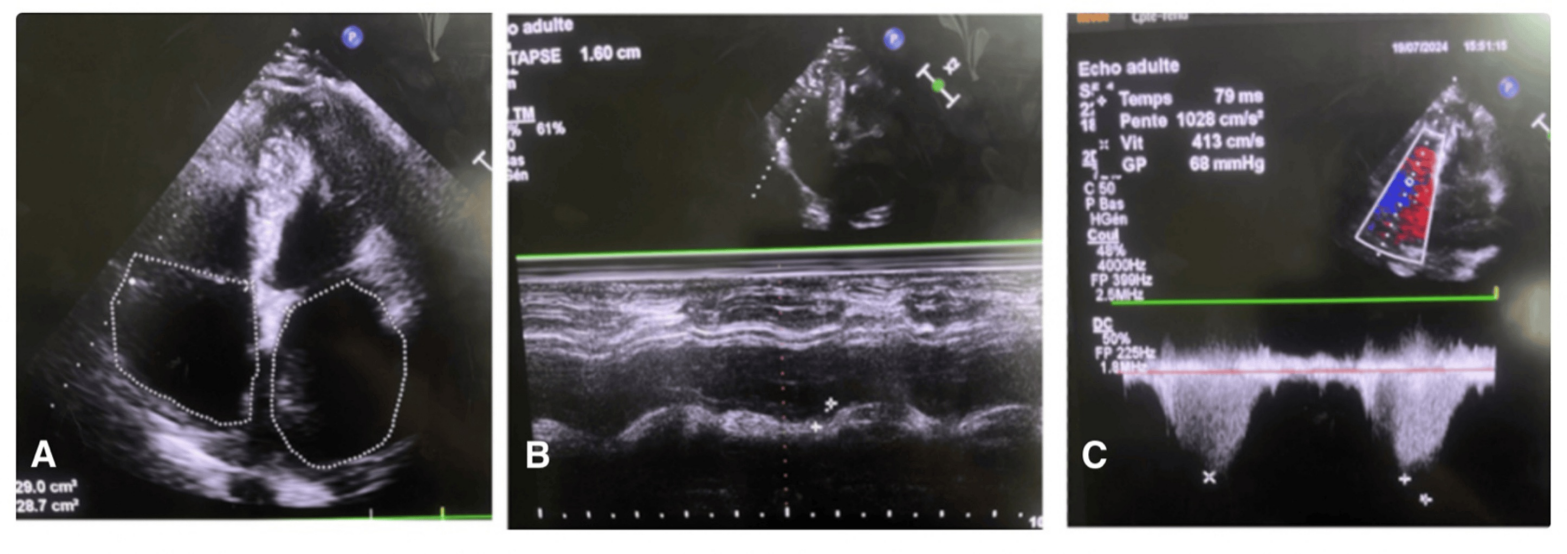
Echocardiographic features. (A) Bilateral atrial dilation. (B) TM-mode demonstrating right ventricular dysfunction. (C) Continuous Doppler assessment demonstrating the presence of significant pulmonary hypertension, with tricuspid regurgitation rated as grade III (Vmax = 4.13 m/s) and an estimated systolic pulmonary artery pressure of 68 + 5 = 73 mmHg.

Bedside echocardiography revealed a large, polylobulated, tissue-like mass measuring 192 × 31 mm (Figure 2), with a broad-based attachment originating from the roof of the left atrium and prolapsing into the left ventricle during diastole (Figure 3). The mass generated a 6 mm gradient between the atrium and the ventricle, causing minimal mitral regurgitation secondary to dilation of the mitral annulus. Severe pulmonary hypertension was also noted, with grade III tricuspid regurgitation, an estimated mean pulmonary arterial pressure of 73 mmHg (68 + 5 mmHg) (Figure 4), and tricuspid annular dilation. The lesion exhibited a smooth, nodular free margin without calcifications, and no pericardial effusion was observed.

**Figure 5:**
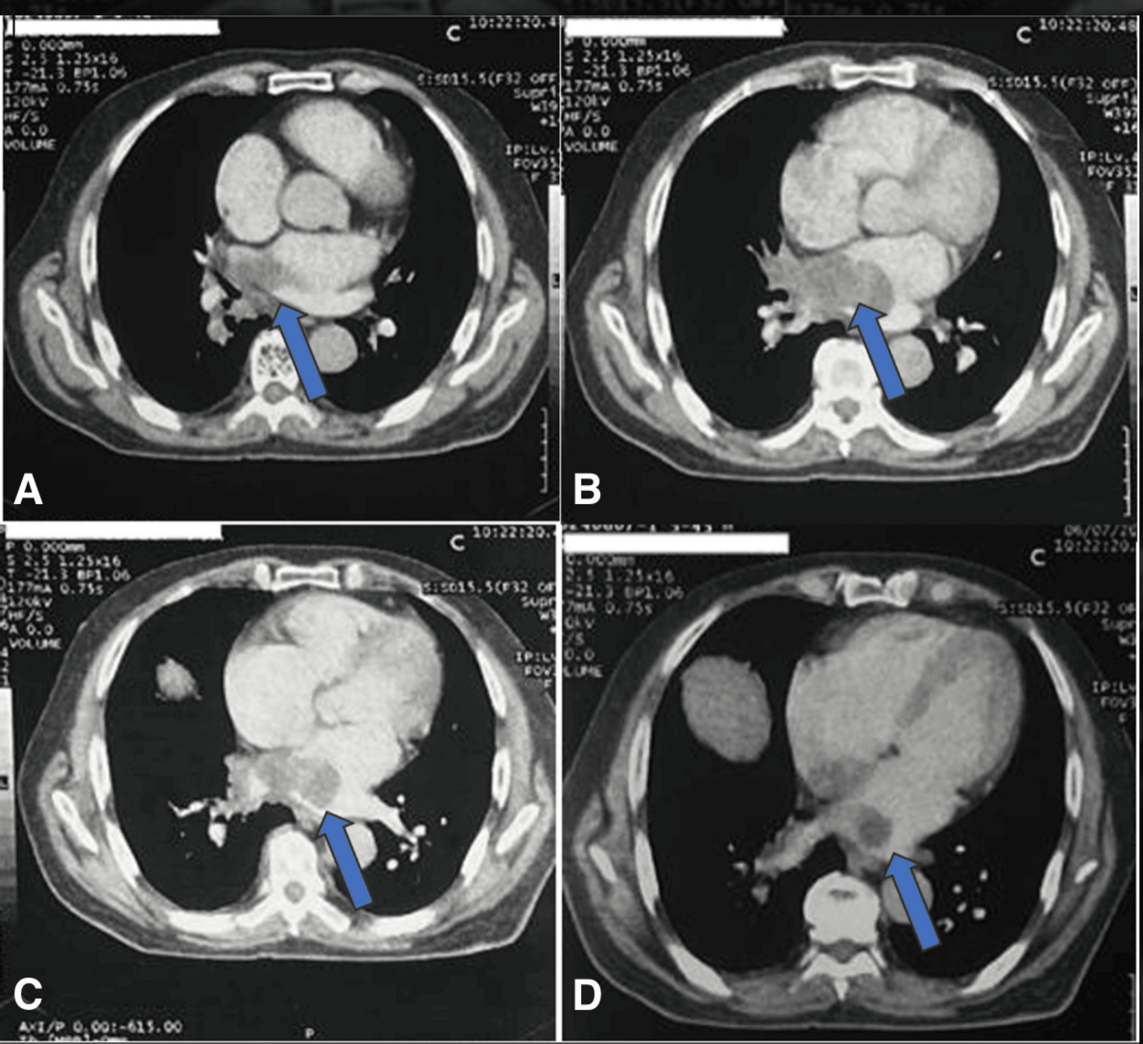
Mediastinal window of the thoracic CT scan demonstrating a multilobular hypodense lesion localized to the posterior wall of the left atrium, extending extraluminally toward the right hilum and the right lower lobe. (A) CT view illustrating the posterior implantation base of the cardiac mass within the left atrium. (B) Multilobulated hypodense appearance of the cardiac mass with hilar extension. (C) Absence of enhancement of the cardiac mass. (D) Anatomical relationships of the cardiac mass with the pulmonary structures.

**Figure 6:**
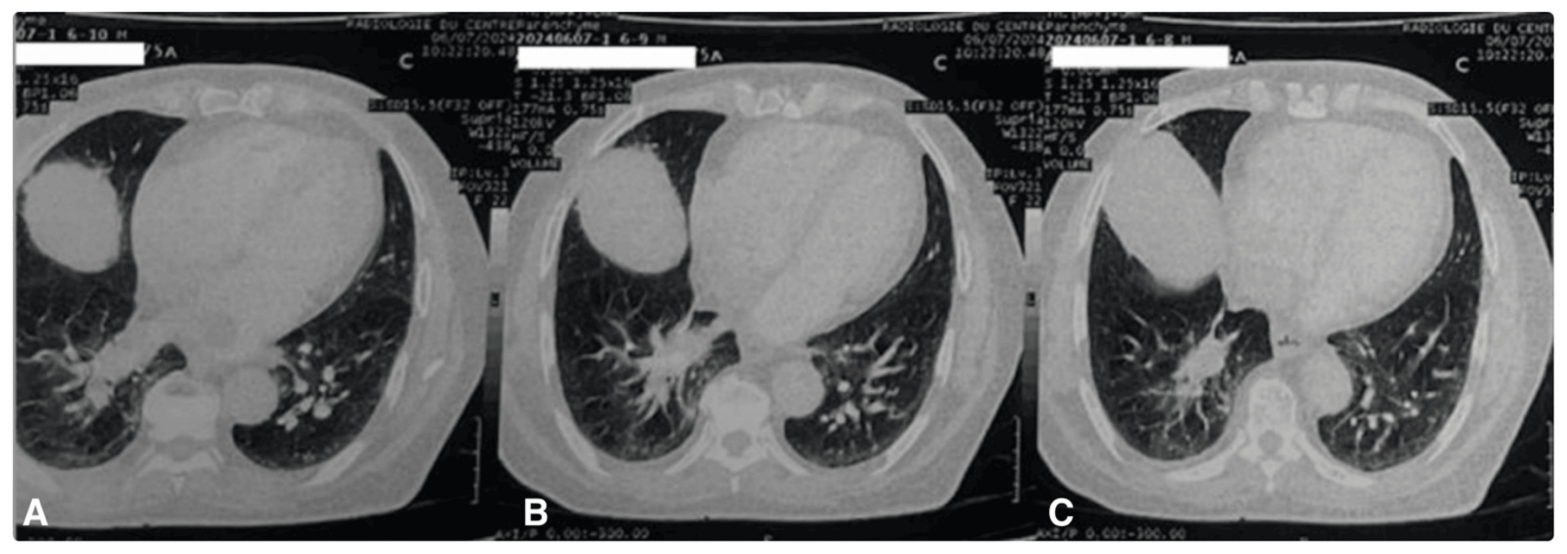
Chest CT in parenchymal window demonstrating parenchymal extension of the cardiac mass into the right lower lobe. (A) Exoluminal component of the cardiac mass extending through the right pulmonary hilum. (B) Extension of the cardiac mass into the right lower lobe parenchyma. (C) CT image revealing parenchymal involvement.

An extension assessment was performed, including a thoracoabdominal pelvic CT scan, which revealed an endoluminal lesion within the left atrium, with a posterior-inferior implantation base. The lesion demonstrated a smooth, nodular free margin and homogeneous hypodensity, without calcifications or significant enhancement. Its exoluminal component extended through the right pulmonary hilum (Figure 5), encasing the middle lobe bronchus and the initial portion of its segmental branches, as well as the right lower lobe (Figure 6), which appeared irregularly narrowed with a small endoluminal projection. Within this hilar infiltration, the right inferior pulmonary vein exhibited a thickened, irregular, and possibly nodular wall, with no enhancement of its afferent branch from Fowler’s vein, suggesting potential thrombosis. Additionally, subcarinal and intercarinal lymphadenopathies measuring 7-8 mm were noted. The liver was of normal size with regular contours and homogeneous density, containing a small 7-mm cyst in segment VI. A nodular thyroid goiter with calcifications was also identified. No other significant abnormalities were detected.

**Figure 7:**
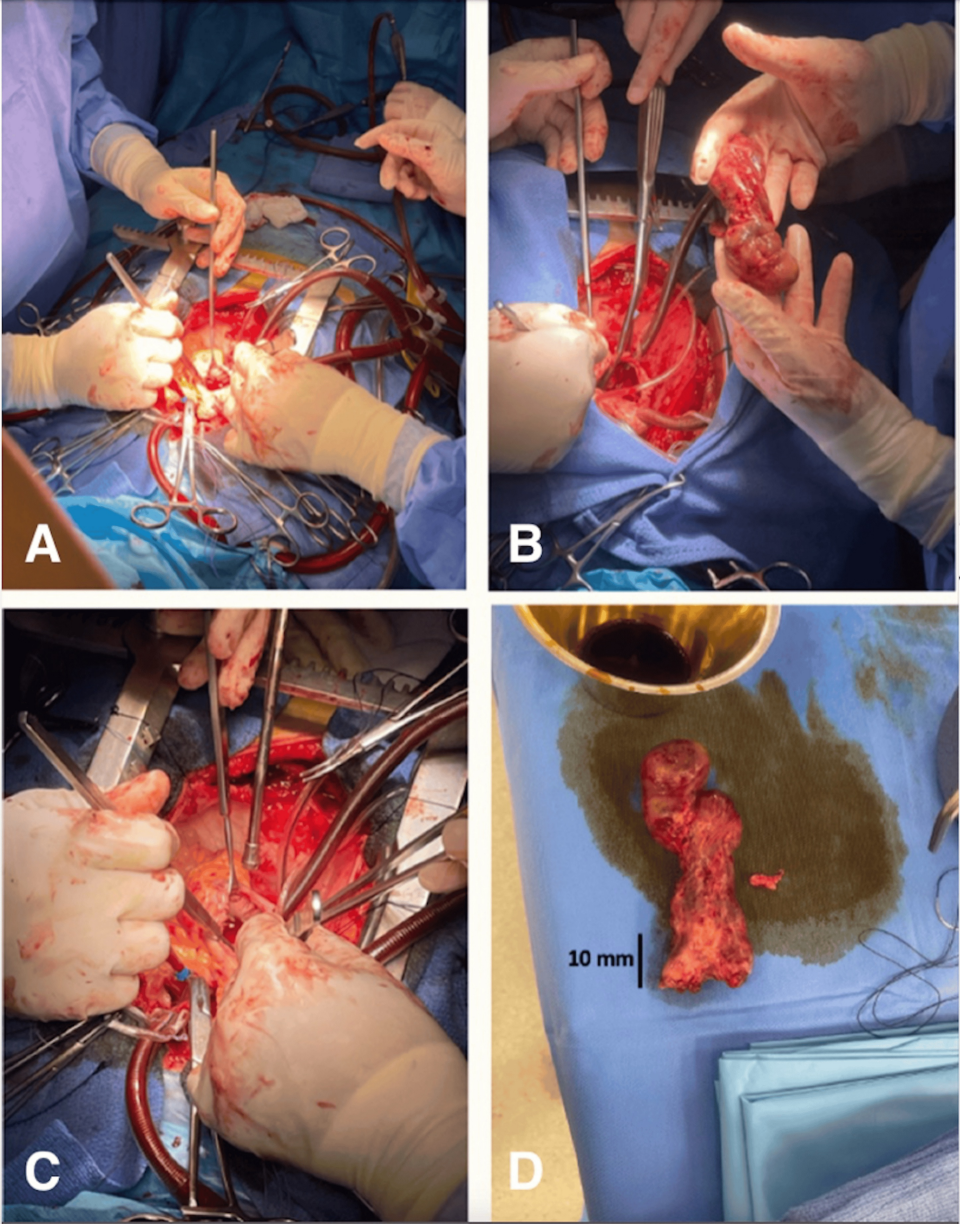
Intraoperative images depicting the tumor resection performed through a median sternotomy, excising a firm, heterogeneous, white tumor with necrotic and hemorrhagic changes, measuring 20 × 8 × 6 cm. (A) Right atriotomy parallel to the atrioventricular sulcus, providing access to the left atrium through a transseptal approach. (B) Excision of the intracardiac mass performed by the cardiovascular surgical team. (C) Final phase of the median sternotomy procedure following the extraction of the mass. (D) Macroscopic aspect of the cardiac mass revealing a heterogeneous, white tumor with necrotic and hemorrhagic changes.

Given the patient’s hemodynamic deterioration, urgent surgical resection of the cardiac mass was undertaken as first-line therapy. The procedure was performed via a median sternotomy. Under cardiopulmonary bypass with aortic cross-clamping, the left atrium was accessed through a transseptal approach, revealing a large tumor measuring 20 × 8 × 6 cm (Figure 7), adherent to the posterior wall of the left atrium at the junction of the pulmonary veins. The mass extended throughout the left atrium and into the left ventricle via the mitral valve, causing obstruction of the left superior pulmonary vein. An en bloc resection of the cardiac mass was performed, yielding a firm, heterogeneous, white tumor with areas of necrosis and hemorrhage. Examination of the mitral valve revealed no significant structural abnormalities or commissural fusion. Intraoperative frozen section analysis indicated a high likelihood of malignancy.

The postoperative course was uneventful, with no significant complications. The patient was gradually weaned off vasoactive agents and remained hemodynamically stable following the intervention. Echocardiography performed on postoperative day five demonstrated a preserved left ventricular ejection fraction and no residual intracardiac tumor.

Histopathological examination revealed a poorly differentiated, extensively altered cellular proliferation. Immunohistochemical analysis confirmed the diagnosis of cardiac rhabdomyosarcoma.

**Figure 8:**
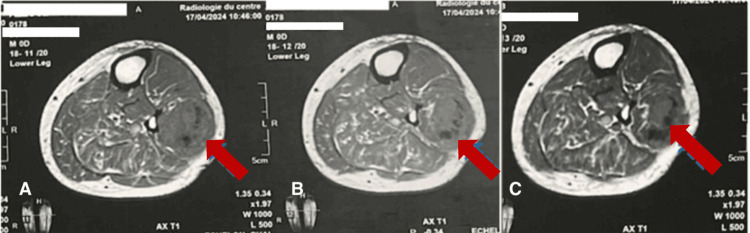
MRI of the left leg in axial T1-weighted sequence demonstrating a lobulated, heterogeneous, indistinctly marginated mass with hemorrhagic changes (dashed arrow). (A-C) Various cross-sectional levels illustrating a rounded, lobulated, and heterogeneous mass measuring 165 × 40 × 50 mm, with necrotic and hemorrhagic alterations.

**Figure 9:**
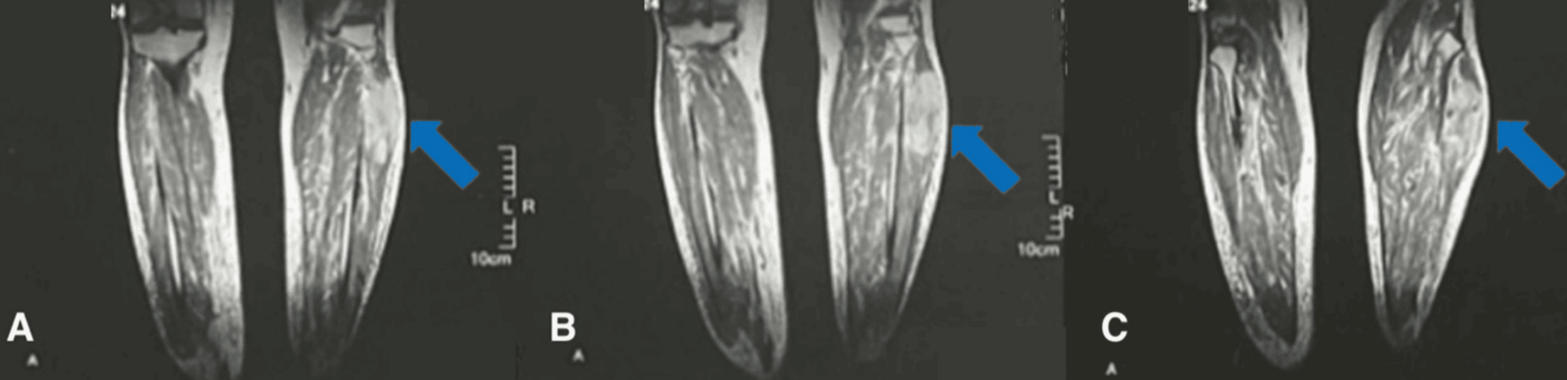
MRI of the left leg in coronal T2-weighted sequence showing a fusiform, heterogeneous mass, measuring 165 × 40 × 50 mm, centered on the fibular muscles in the upper third of the left leg (arrow). (A-C) Various coronal sections demonstrating a fusiform mass centered on the fibular muscles.

Further staging was completed with an MRI of the left leg to better characterize the clinically palpable mass, as well as positron emission tomography (PET) scanning. The MRI demonstrated a fusiform, lobulated, and heterogeneous mass measuring 165 × 40 × 50 mm, centered on the fibular muscles in the upper third of the left leg, highly suggestive of a sarcomatous lesion (Figures 8, 9).

The scan revealed multiple hypermetabolic foci, including a mass in the soft tissues of the upper lateral third of the left leg, adjacent to the fibular diaphysis, an intracardiac hypermetabolic lesion, and increased uptake in the right lung, external iliac lymph nodes, bones, pelvic soft tissues, and lower extremities.

Based on these findings, a biopsy of the leg lesion was performed, demonstrating an aggressive fusocellular mesenchymal tumor. Immunohistochemical analysis confirmed its rhabdomyosarcomatous nature.

The diagnosis was established as fibular rhabdomyosarcoma with metastatic involvement of the heart and bronchial extension via the hilar plate. Notably, the patient had first presented with the leg lesion 11 months before admission, whereas the most recent echocardiogram, performed eight months before admission, had shown no significant abnormalities, including no evidence of an intracardiac mass in the left chambers.

The patient was discharged 10 days postoperatively and subsequently referred to the oncology team for further management. A multidisciplinary case conference recommended an adjuvant chemoradiotherapy regimen, including vincristine, epirubicin, and cyclophosphamide, targeting the metastatic sites to reduce recurrence risk and improve overall survival.

## Discussion

Cardiac tumors constitute a rare pathological entity, encompassing a diverse array of neoplasms mostly classified into primary and secondary tumors. Although primary cardiac tumors are infrequent, with an average incidence ranging of 0.001% to 0.03%, cardiac metastases are, in reality, more prevalent than might be assumed, with an estimated incidence of 2.3% to 18.3% [2].

In fact, primary cardiac tumors are infrequently encountered and are predominantly benign, with myxomas being the most common. However, malignant tumors constitute approximately 25% of all primary cardiac tumors, primarily comprising sarcomas. Although rhabdomyosarcoma is rare, it represents the second most prevalent histological subtype of primary cardiac sarcomas, following angiosarcoma, and accounts for 20% of all cardiac sarcomas [3].

Conversely, cardiac metastases are more frequently documented in the literature, often resulting from a multi-metastatic disease that has disseminated to multiple organs by the time cardiac involvement is identified. The most commonly involved primary malignancies include lung cancer, breast cancer, malignant melanoma, and lymphoma.

Nevertheless, secondary cardiac localization of soft tissue rhabdomyosarcoma remains exceedingly rare. In fact, sarcomas remain a very uncommon neoplasm representing fewer than 1% of adult malignancies. Rhabdomyosarcoma is an aggressive subtype that originates from mesenchymal tissue and has a high metastatic potential. It is composed of immature, incompletely differentiated striated muscle cells. We reported the case of a 75-year-old male patient with metastatic rhabdomyosarcoma originating from the fibular muscles, which metastasized to the left atrium, with extension to the left ventricle and the bronchi. This exceptionally rare case highlights the aggressive nature of rhabdomyosarcoma and its propensity for rapid dissemination.

Indicative symptoms of cardiac localization are non-specific and can vary widely, including rapidly worsening dyspnea, episodes of palpitations potentially due to arrhythmias such as atrial fibrillation or atrial flutter, conduction disturbances such as high-degree atrioventricular block, chest pain, murmurs suggestive of mitral regurgitation or mitral stenosis, signs of acute heart failure, pericardial effusion, or even tamponade. Moreover, due to its substantial locoregional aggressiveness and metastatic potential, rhabdomyosarcoma may extend through the pulmonary hilum and invade adjacent lung regions, thereby leading to respiratory symptoms, as observed in our patient.

Although clinical patterns are diverse, symptoms of cardiac metastasis may remain undetected for an extended period until the tumor occupies substantial space within the cardiac chambers and extensively invades the myocardium. In fact, the clinical presentation is directly associated with the tumor’s site of implantation and proportionate to the extent of myocardial infiltration [4].

The diagnosis of a cardiac mass relies on imaging studies, with transthoracic echocardiography serving as the first diagnostic tool. This technique enables the visualization of the mass, as well as the assessment of its size, shape, mobility, site of implantation, and hemodynamic impact on the heart. However, transthoracic echocardiography does not provide detailed information about the malignant or benign nature of the mass. CT of the chest and cardiac MRI are crucial for further evaluating the suspicion of malignancy, as they offer detailed insights into the characteristics, precise topography, and locoregional extent of the mass. Furthermore, CT plays a crucial role in the staging of the tumor by identifying additional secondary sites [5].

Similarly, PET is useful for detecting areas of increased radiotracer uptake, providing detailed localization of various lesions and offering insights into their malignant potential [6]. A definitive diagnosis concerning the histological nature of the tumor is established exclusively through histopathological examination combined with immunohistochemical analysis [7]. In the context of rhabdomyosarcoma, pronounced positivity for anti-desmin antibodies is highly specific for confirming the diagnosis, as evidenced by the histopathological findings in both the leg mass and the intracardiac surgical specimen from our patient.

The prognosis for rhabdomyosarcoma remains markedly bleak, particularly in cases with metastatic presentations. Although there have been reports of survival extending up to five years [9], the overall prognosis remains exceedingly unfavorable. Patients generally have a median survival of less than 12 months [8-10].

Data in the literature regarding therapeutic strategies are still limited and lack standardization. There is currently no clear consensus regarding the therapeutic approach for metastatic rhabdomyosarcoma involving cardiac localizations, and the efficacy of radiochemotherapy remains uncertain. However, multimodal management, which includes radiochemotherapy with or without surgical intervention, is presently considered the standard treatment approach. The optimal therapeutic strategy involves surgical resection of the intracardiac mass, complemented by adjuvant radiochemotherapy, depending on the tumor’s operability and the patient’s overall health status. [9] In our case, we used an anthracycline-based chemotherapy combination, including vincristine, epirubicin, and cyclophosphamide.

Regardless of the poor prognosis associated with patients presenting with metastatic rhabdomyosarcoma, surgical intervention is warranted to excise the mass, alleviate acute symptoms, and potentially extend the patient’s life by several months.

In our patient’s case, resection of the cardiac mass was followed by notable clinical improvement. The patient is currently asymptomatic from a cardiovascular standpoint and is about to begin an adjuvant radiochemotherapy regimen aiming to enhance his life expectancy.

## Conclusions

Rhabdomyosarcoma, although intrinsically rare, is an exceptionally aggressive tumor characterized by rapid growth, high propensity for recurrence, and significant metastatic potential. Cardiac metastasis from soft tissue rhabdomyosarcoma is exceedingly uncommon, making this case a particularly noteworthy example in the medical literature. The non-specific nature of symptoms associated with cardiac involvement often delays diagnosis until substantial myocardial infiltration has occurred, highlighting the critical role of advanced imaging modalities. Histopathological analysis remains essential for establishing a definitive diagnosis and confirming the histological subtype. Although the prognosis remains challenging, surgical intervention can provide symptomatic relief and may contribute to prolonging survival in selected cases; however, such claims should be interpreted cautiously, given the limited long-term outcome data. This case underscores the rare and aggressive nature of cardiac metastasis from soft tissue rhabdomyosarcoma and provides valuable insights into its clinical management. A personalized, multidisciplinary approach is essential to facilitate early diagnosis, optimize therapeutic strategies, and, ultimately, improve patient outcomes.
